# A molecular nematic liquid crystalline material for high-performance organic photovoltaics

**DOI:** 10.1038/ncomms7013

**Published:** 2015-01-14

**Authors:** Kuan Sun, Zeyun Xiao, Shirong Lu, Wojciech Zajaczkowski, Wojciech Pisula, Eric Hanssen, Jonathan M. White, Rachel M. Williamson, Jegadesan Subbiah, Jianyong Ouyang, Andrew B. Holmes, Wallace W.H. Wong, David J. Jones

**Affiliations:** 1 School of Chemistry, Bio21 Institute, The University of Melbourne, 30 Flemington Road, Parkville, Victoria 3010, Australia; 2 Department of Materials Science and Engineering, National University of Singapore, 7 Engineering Drive 1, Singapore 117574, Singapore; 3 Department of Renewable Energy, School of Power Engineering, Chongqing University, 174 Shazhengjie, Shapingba, Chongqing 400044, China; 4 Max Planck Institute for Polymer Research, Ackermannweg 10, 55128 Mainz, Germany; 5 Advanced Microscopy Facility, Bio21 Institute, The University of Melbourne, 30 Flemington Road, Parkville, Victoria 3010, Australia; 6 MX Beamlines, Australian Synchrotron, 800 Blackburn Road, Clayton, Victoria 3168, Australia

## Abstract

Solution-processed organic photovoltaic cells (OPVs) hold great promise to enable roll-to-roll printing of environmentally friendly, mechanically flexible and cost-effective photovoltaic devices. Nevertheless, many high-performing systems show best power conversion efficiencies (PCEs) with a thin active layer (thickness is ~100 nm) that is difficult to translate to roll-to-roll processing with high reproducibility. Here we report a new molecular donor, benzodithiophene terthiophene rhodanine (BTR), which exhibits good processability, nematic liquid crystalline behaviour and excellent optoelectronic properties. A maximum PCE of 9.3% is achieved under AM 1.5G solar irradiation, with fill factor reaching 77%, rarely achieved in solution-processed OPVs. Particularly promising is the fact that BTR-based devices with active layer thicknesses up to 400 nm can still afford high fill factor of ~70% and high PCE of ~8%. Together, the results suggest, with better device architectures for longer device lifetime, BTR is an ideal candidate for mass production of OPVs.

## Abstract (web summary)

There is a trade-off between increasing thickness of active layers in organic photovoltaic cells to be compatible with modern printing techniques and decreasing it to improve the device performance. Sun *et al.* report a nematic liquid crystalline molecular electron donor material used in thick layers.

## Graphical abstract

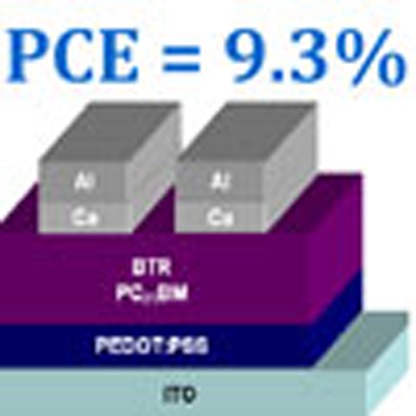

Despite recent developments in solid-state photovoltaic devices^1^,^2^,^3^, bulk-heterojunction (BHJ) organic photovoltaics (OPVs)^4^ continue to be a promising low-cost renewable energy technology. The reasons for this outlook include the versatility of organic semiconducting materials and simple device architectures that can be constructed from a variety of printing techniques. The development of the BHJ OPVs has been rapid in recent years driven by a combination of organic material design, interface engineering and improvements in device geometry. The reported power conversion efficiency (PCE) of single-junction small-area devices is now routinely in the 6–8% range^5^,^6^,^7^.

Published reports of single-junction BHJ OPVs over 9% PCE are still rare. A handful of polymeric electron donor materials and only one molecular donor have been reported in devices that reached this benchmark^8^,^9^,^10^,^11^,^12^,^13^. Molecular OPVs are an attractive alternative to polymer-based OPVs. Higher material purity can be achieved with the well-defined discrete structure of molecules and this should ensure greater reproducibility in devices^14^,^15^,^16^. To achieve high photovoltaic conversion efficiency, the material should be capable of forming good films with high molecular order. This can be achieved by smart molecular design and control over crystallization processes. For molecular semiconductors, conjugated flat and rigid backbones are preferred for easy packing via π–π interactions. Good solubility is conferred through employment of the appropriate number, type and length of side chains, without hindering the packing of the backbones. The desirable donor phase in BHJ OPVs needs well-ordered nanocrystals with sizes comparable to the exciton diffusion length for efficient charge generation. Strategies simultaneously to enhance molecular order and restrict crystal size have been reported, including thermal annealing, solvent additives, solid additives and solvent vapour annealing (SVA)^7^,^17^,^18^,^19^,^20^,^21^,^22^. Recent reports showed that the rapid SVA treatment was particularly useful in achieving high fill factor (FF) and PCE in molecular OPVs^23^,^24^. Solvent selection rules for SVA treatment were identified in our previous study^23^.

Despite the important progress achieved in small-area OPV devices fabricated in laboratories, the successful commercialization of OPV technology relies on the application of solution-processed roll-to-roll techniques for large-scale printing^25^. One of the challenges in printing OPV devices is in printing the optimal active layer thickness of 80–120 nm that many of the high-performing material systems require, while obtaining pinhole-free thin films reproducibly at high printing speed. This problem can be relieved by printing thick films with thickness over 200 nm^26^. Unfortunately, due to limited charge diffusion length, thick-film OPV devices often experience severe bimolecular recombination and space charge effect, leading to reduced FF and PCE^27^. So far only a few studies have achieved high photovoltaic performance on polymer-based OPVs, with active layers above 200 nm^26^,^28^,^29^,^30^. No report has been found for molecular OPVs.

In this work, a new molecular electron donor material, benzodithiophene terthiophene rhodanine (BTR), with a benzo[1,2-*b*:4,5-*b*′]dithiophene (BDT) core and rhodanine peripheral units was developed and used in OPV devices giving PCEs >9%. While its π-conjugated structure is analogous to a high-performance compound reported previously^7^,^31^, the strategic placement of the side chains provided BTR with strong intermolecular interactions, as evidenced by its liquid crystalline (LC) behaviour. Such interactions translated successfully into excellent hole transport properties; hole mobilities up to 0.1 and 1.6 × 10^−3^ cm^2^ V^−1^ s^−1^ were recorded by organic field-effect transistor (OFET) and space-charge-limited current (SCLC) methods, respectively. Thus, BTR-based OPVs with thick active layers (300–400 nm) could still afford PCEs of over 8% with high FF of ~70%. Normal cell architecture employed in this study showed a moderate device lifetime. With better cell architectures or proper encapsulation for longer device lifetime, it is believed that BTR is a very attractive candidate for roll-to-roll printed OPV modules.

## Results

### Physical properties of BTR

**Figure 1:**
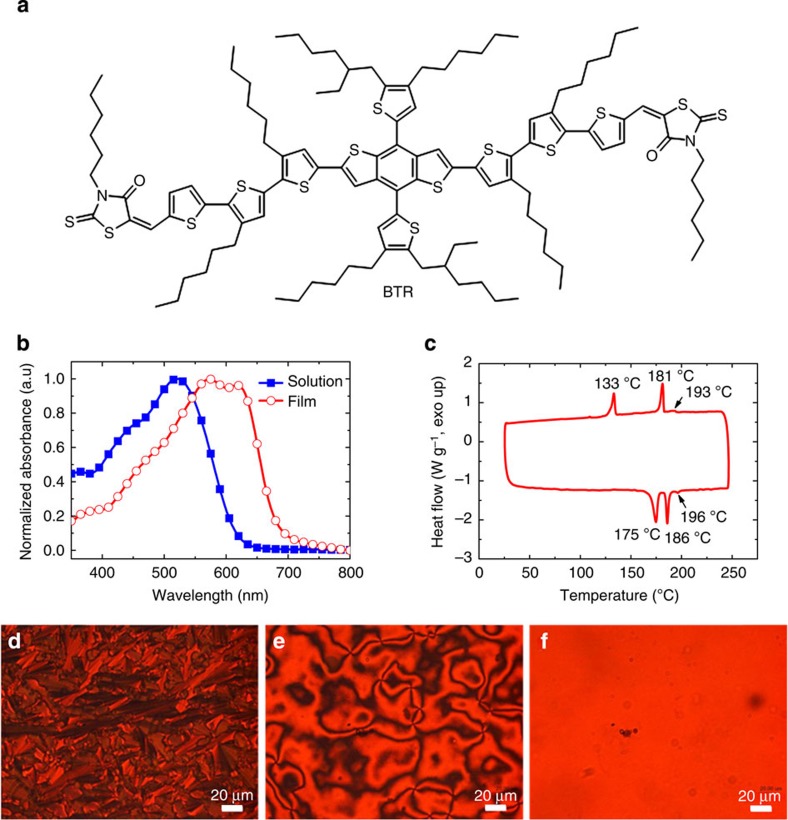
BTR chemical structure and physical properties. (**a**) Chemical structure of BTR. (**b**) Normalized UV–vis absorption spectra of BTR in chloroform (5 mg ml^−1^) and in a spin-cast film. (**c**) DSC thermogram of BTR in nitrogen at a ramp rate of 10 °C min^−1^. The lower trace is from the heating cycle and upper trace from the cooling cycle. (**d**) BTR thin film sandwiched in between two glass slides observed under a polarized optical microscope (POM) at a stage temperature of 185 °C. (**e**) The POM image of the same BTR thin film at the same settings when the stage temperature rises to 195 °C. (**f**) The POM image taken at a stage temperature of 197 °C.

The BTR molecule was synthesized in two steps from known precursors in a good yield (Supplementary Fig. 1). The chemical structure of BTR is shown in Fig. 1a. The backbone consisting of the BDT unit, two terthiophenes and two rhodanine groups formed a coplanar structure. In comparison with analogous structures in the literature^7^,^31^, the side chains of BTR were shortened and positioned at the terthiophene building blocks in a regioregular manner to facilitate side-chain interdigitation^32^,^33^. In combination with the additional hexyl group on the thienyl-BDT unit, the side chains of BTR imparted LC behaviour (vide infra) that was not observed in previous reports.

BTR shows an excellent solubility of 211 mg ml^−1^ in chloroform, as derived from concentration and absorption data (Supplementary Fig. 2). BTR in solution displays an absorption maximum (*λ*_max_) at 523 nm, with an extinction coefficient (*ε*) of 1.10 × 10^5^ M^−1^ cm^−1^ (Fig. 1b; Supplementary Table 1). The high *ε* is attributed to the planarity of its backbone. The BTR solid film exhibits a red-shift of the *λ*_max_ to 572 nm relative to that in solution. Furthermore, an additional absorption peak at 620 nm appears in the absorption spectrum of a thin film. The red-shift and new absorption peak of the BTR film suggest the presence of strong intermolecular interaction and aggregation in the solid film. The absorption onset of the BTR film is at 681 nm, equivalent to an optical frontier orbital energy gap of 1.82 eV. Determined by cyclic voltammetry (CV) (Supplementary Fig. 3), the highest occupied molecular orbital (HOMO) and lowest unoccupied molecular orbital (LUMO) of BTR are −5.34 and −3.52 eV, respectively. The HOMO–LUMO gap of BTR is 1.82 eV, which is in good agreement with the optical energy gap. Because the open-circuit voltage (*V*_oc_) is largely determined by the HOMO–LUMO gap of the donor and acceptor, a deep-lying HOMO of BTR can potentially support a high *V*_oc_. In combination with fullerene acceptor [6,6]-phenyl C_71_ butyric acid methyl ester (PC_71_BM), whose LUMO level is around −4.0 eV, the LUMO energy offset of 0.48 eV between BTR and PC_71_BM should provide enough driving force for exciton dissociation^34^.

The BTR molecule has good thermal stability with a decomposition temperature of 405 °C in nitrogen (5% weight loss in thermogravimetric analysis, Supplementary Fig. 4). BTR exhibits a sharp differential scanning calorimetry (DSC) peak at 175 °C (Fig. 1c), which is assigned to secondary crystalline phase transition by means of a structural analysis (vide infra). Furthermore, a melting temperature of 186 °C into a LC phase and a clearing temperature at 196 °C of small enthalpy into an isotropic melt were observed. Upon cooling, three exothermic peaks at 193, 181 and 133 °C were recorded. The first minor transition was attributed to the LC phase transition, while the two major ones were related to the crystallization process of the two crystalline phases. To observe directly the LC transition and precisely assign the phases, BTR powder was sandwiched in between two glass slides, heated and examined under a polarized optical microscope (POM). The BTR molecule was highly crystalline below a stage temperature of 185 °C (Fig. 1d). Between 185 and 195 °C, the crystalline solid was replaced by a liquid crystal nematic texture (Fig. 1e). The nematic phase suggests that BTR molecules have a rigid rod-like shape, which can maintain a long-range directional order with their long axes parallelly aligned. They can thus have high crystallinity in solid state^17^,^35^,^36^,^37^. The liquid crystal transformed above 196 °C into an isotropic melt, leaving no prominent feature under the POM (Fig. 1f). Thereby, the small transition enthalpy determined by DSC is in agreement with the low-ordered nematic phase. The nematic LC behaviour is an important feature of the BTR molecule, implying strong intermolecular interaction resulted from side-chain modifications, and potentially high charge carrier mobility due to three-dimensional (3D) charge transport^38^.

### Crystal packing of BTR molecules

**Figure 2:**
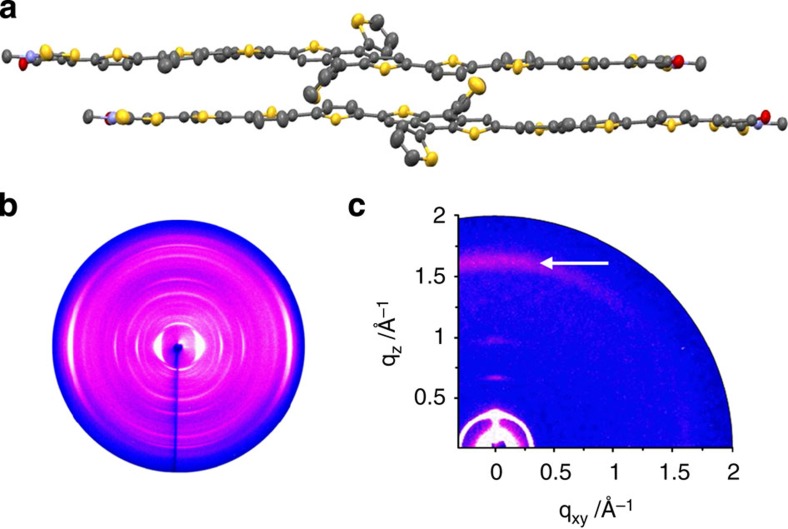
Crystal packing resolved by X-ray techniques. (**a**) Centrosymmetric π-stacked dimers of BTR molecules in its single crystal, the alkyl side chains have been omitted for clarity. (**b**) 2D-WAXS of BTR filament measured at 30 °C. (**c**) GIWAXS of the as-cast BTR thin film on silicon wafer via spin coating (π-stacking reflection is indicated by an arrow).

To obtain a better understanding of the packing of BTR molecules in the solid, X-ray quality single crystals of BTR were grown from a mixed solution of 2-propanol and dichloromethane by slow evaporation. The single crystal structure was solved using data from the MX2 beamline at the Australian Synchrotron^39^ (Fig. 2a; Supplementary Figs 5–8). The X-ray crystal structure of the BTR molecule revealed a coplanar structure of the conjugated backbone, which should facilitate light absorption and also crystal stacking. The crystal packing is dominated by π-stacking between the individual BTR backbones that arrange themselves into π-stacked centrosymmetric dimers with an average inter-plane separation of ca 3.60 Å (Fig. 2a). These individual dimers aggregate together by π-stacking, with an average interplaner separation of 3.62 Å (Supplementary Fig. 6). This type of packing is consistent with the bathrochromic shifting of the absorption from solution to the solid film (*J*-aggregate).

The solid-state structure was also examined using two-dimensional wide-angle X-ray scattering (2D-WAXS) on neat BTR filaments. The samples were prepared by filament extrusion^40^, which imparted bulk orientation on the crystalline material. The 2D-WAXS pattern suggests a crystalline character of BTR in the low-temperature phase as evident by the high number of distinct reflections (Fig. 2b). The molecules are organized in a layered structure that is aligned in the direction of the fibre axis. An interlayer distance of 18.3 Å is determined from reflections located in the equatorial small-angle range. On the same plane of the pattern, two π-stacking peaks appear that are related to distances of 3.70 and 3.65 Å of stacked BTR dimers. These values are in the same range as found for the single crystal. Further meridional reflections are originated from intramolecular correlations along the extended conjugated BTR backbone. At 179 °C, the sample maintains a crystalline phase, however, with a slightly smaller degree of order (Supplementary Fig. 9). The interlayer spacing remains identical at 18.3 Å, while only one and a little larger stacking distance was observed at 3.76 Å.

In a thin solid film, BTR organizes in two different molecular arrangements as indicated by the grazing incidence wide-angle X-ray scattering (GIWAXS) pattern in Fig. 2c. Reflections in the meridional plane (along *q*_xy_=0 Å^−1^) in the small- and middle-range scattering region are related to the formation of a layered structure with an interlayer distance of 18.7 Å. In addition, 3rd-order reflections are visible typical for a long-range order, while their position on the meridional plane of the pattern is characteristic for an edge-on molecular organization. In this arrangement, the backbone plane is aligned perpendicular to the surface. However, the corresponding equatorial π-stacking peak of the edge-on arranged molecules assembled in the layered structure is too weak to be detected. Instead, the π-stacking reflection related to a single distance of 3.70 Å is located also on the meridional plane, which is typical for a face-on arrangement. These results imply two distinct surface organizations. In the first phase, the molecules are π-stacked and face-on arranged, but do not organize in a layered structure. In the second phase, the molecules are edge-on aligned with respect to the substrate, but are disordered within the layer organization. Because charge transport in organic semiconductors is mainly via hopping between adjacent molecules, the co-existence of edge-on and face-on orientations can potentially form a 3D network for hopping, thus beneficial to charge transport^41^.

### OFET mobility

To study the charge carrier transport, OFETs using different procedures were built. For top-contact devices, BTR was spin-coated from a 4.5-mg ml^−1^ toluene solutionand subsequently annealed at 179 °C. These transistors delivered hole mobilities up to 0.01 cm^2^ V^−1^ s^−1^ (Supplementary Fig. 10). Bottom-contact OFET devices with the BTR molecules deposited by drop-casting gave mobility values as high as 0.1 cm^2^ V^−1^ s^−1^ (Supplementary Fig. 11). It should be noted that the OFET devices were not intensively optimized. The primary purpose of the OFET experiments was to show the potential of the BTR material as a semiconductor.

### Photovoltaic performances

**Figure 3:**
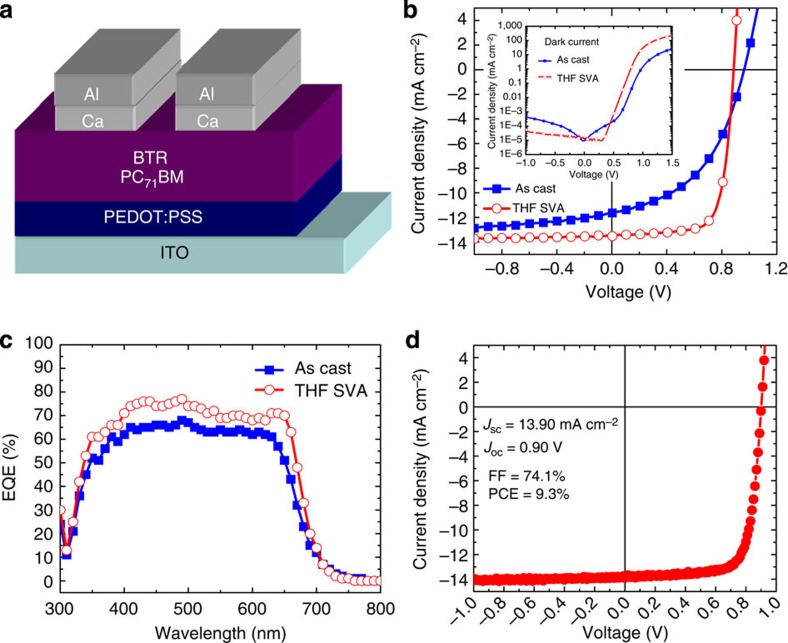
Device architecture and photovoltaic performances. (**a**) Schematic diagram of a normal cell architecture used in this study. (**b**) *J–V* characteristics of BTR:PC_71_BM BHJ solar cells with or without THF solvent vapour annealing tested in air under 98 mW cm^−2^ AM1.5G illumination. Inset: dark current plotted in a semi-log scale of the two solar cells. (**c**) EQE spectra of optimized BTR-based solar cells with or without THF SVA treatment. (**d**) *J–V* curve of the most efficient BTR:PC_71_BM BHJ solar cell after 15 s of THF SVA measured by an independent research institute in nitrogen atmosphere under an illumination of 100 mW cm^−2^.

The excellent solubility, strong intermolecular interaction, suitable absorption profile and energy levels, as well as encouraging semiconducting properties prompted us to explore the photovoltaic performance of the BTR molecule. The OPV cells adopted a simple normal architecture, with the BTR:PC_71_BM blend film sandwiched between a poly(3,4-ethylenedioxythiophene):poly(styrenesulfonate) (PEDOT:PSS)-coated indium tin oxide (ITO) transparent anode and a Ca/Al back cathode (Fig. 3a). We further treated the active layer with SVA, which has been shown to be effective in enhancing the performance of molecular OPVs^22^,^23^,^24^. The SVA treatment was carried out by exposing the as-cast active layer to solvent vapours. According to the solvent selection rules previously identified^23^, tetrahydrofuran (THF) was chosen for SVA owing to the moderate solubility of BTR in THF (89 mg ml^−1^).

The BTR-based OPVs with an optimal active layer thickness of 250 nm were encapsulated and tested in air. The current density (*J*)–voltage (*V*) curves of the best devices are shown in Fig. 3b, with the photovoltaic parameters summarized in Table 1. Without SVA treatment, the highest performance for the as-cast OPVs showed short-circuit current density (*J*_sc_)=11.64 mA cm^−2^, *V*_oc_=0.96 V, FF=47% and PCE=5.2%. SVA treatment significantly enhanced the photovoltaic performance. OPVs with 15 s of THF SVA exhibited *J*_sc_=13.52 mA cm^−2^, *V*_oc_=0.89 V, FF=73% and PCE=8.7%. Device assembly was reproducible with around 60 SVA-treated OPV devices having an average PCE of 8.3±0.2%. Thermal annealing was found to diminish the device performance, due to the overgrowth of the phases (Supplementary Fig. 12).

The causes for the enhanced FF after SVA treatment were investigated by measuring dark currents (inset of Fig. 3b). Compared with an as-cast molecular OPV, the SVA-treated sample displayed notably higher current density under positive bias. In great contrast, the current density was one order of magnitude smaller in reverse bias. To further understand the SVA treatment effect, series resistance (*R*_s_) and shunt resistance (*R*_sh_) were extracted at 1.5 and 0 V of the dark curves (Table 1). Without SVA treatment, the OPV had a *R*_s_ of 14.0 Ω cm^2^ and a *R*_sh_ of 5.5 MΩ cm^2^. SVA treatment led to a reduction of *R*_s_ by six times and a slight increase of *R*_sh_. Together, the results suggest the SVA treatment can suppress leakage current and improve the diode behaviour.

The slight improvement in *J*_sc_ after SVA treatment was monitored by external quantum efficiency (EQE) measurement (Fig. 3c). A high EQE of over 60% was measured in the visible region from 400 to 650 nm for the non-annealed OPV. The *J*_sc_ calculated by integrating the product of photon flux and EQE at each wavelength was 11.70 mA cm^−2^, which was in good agreement with the measured *J*_sc_ (11.64 mA cm^−2^). The SVA treatment lifted the EQE in the entire absorption range. In particular, the EQE stayed above 70% between 400 and 650 nm, and a shoulder was found at 640 nm. As a result, the calculated *J*_sc_ increased to 13.53 mA cm^−2^. The EQE result clearly indicates SVA treatment plays a positive role in charge generation, transport and/or collection.

Bearing in mind that OPVs with normal cell architecture are not stable in air, we fabricated a batch of 20 devices in Singapore and 8 devices in Australia and tested them under inert atmosphere using the facilities at Solar Energy Research Institute of Singapore and the Commonwealth Scientific and Industrial Research Organisation, respectively. The best BTR-based OPV fabricated in Singapore exhibited a record efficiency of 9.3%, with *J*_sc_=13.90 mA cm^−2^, *V*_oc_=0.90 V and FF=74.1% (Fig. 3d; Table 1). The results were highly reproducible. The same PCE of 9.3% with a *J*_*sc*_ of 13.40 mA cm^−2^, a *V*_oc_ of 0.90 V and an extremely high FF of 77.0% was achieved in Australia (Table 1). This result demonstrates molecular OPVs can achieve comparable efficiencies attainable by polymer-based OPVs^8^,^9^,^10^,^11^. It is worth noting that the FF of 77.0% is among the highest FF value reported in the literature for solution-processed molecular OPVs^12^,^42^. The average photovoltaic parameters for the 28 devices were *J*_sc_=13.49±0.28 mA cm^−2^, *V*_oc_=0.89±0.01 V, FF=74±1% and PCE=8.9±0.2% (Table 1).

### OPVs of a thick active layer

**Figure 4:**
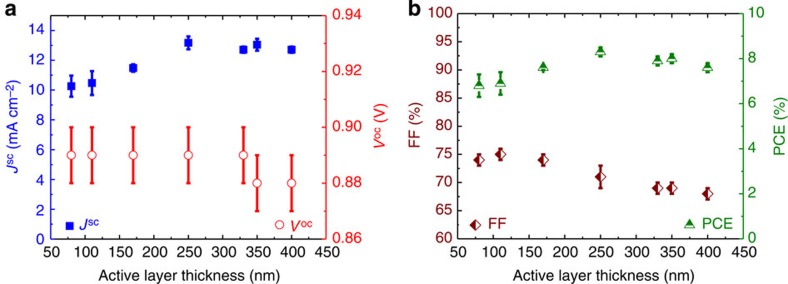
Active layer thickness-dependent variation of photovoltaic performances. (**a**) Plots of *J*_sc_ or *V*_oc_ vs active layer thickness ranging from 80 to 400 nm. (**b**) Plots of FF or PCE against active layer thickness. The results are an average value of >8 devices. The error bars represent the standard deviation from >8 devices.

The high FF values suggest that the BTR-based OPVs can accommodate a greater range of active layer thicknesses. This is particularly important in roll-to-roll printing of very thin films, which are difficult to be precisely controlled, and pinholes are often found in thin-film devices. We were motivated to explore the thickness-dependent solar cell performance using the BTR molecule. Active layers with different thicknesses ranging from 80 to 400 nm were fabricated by tuning the solution concentrations and spin rates. Figure 4 and Supplementary Table 2 show that BTR-based OPVs maintain a nearly constant *V*_oc_ between 0.87 and 0.90 V. The average *J*_sc_ increases from ~10 to ~13 mA cm^−2^ as the active layer thickness increases from 80 to 250 nm and then it saturates around 13 mA cm^−2^ when the thickness further increases to 400 nm. Surprisingly, the FF values for BTR-based OPVs remain high and close to 70% even at thicknesses up to 400 nm. This is not commonly observed in thick-film OPVs, whether it is a molecular OPV or a polymer-based solar cell^26^,^27^,^28^,^29^,^43^. As a result, the overall PCEs formed a flat bell curve with a minimum average value of 6.8% and maximum average value of 8.3% at an active layer thickness of 250 nm. The large tolerance for the active layer thickness makes the BTR molecule a strong candidate for printed OPVs.

### Solvent vapour annealing

**Figure 5:**
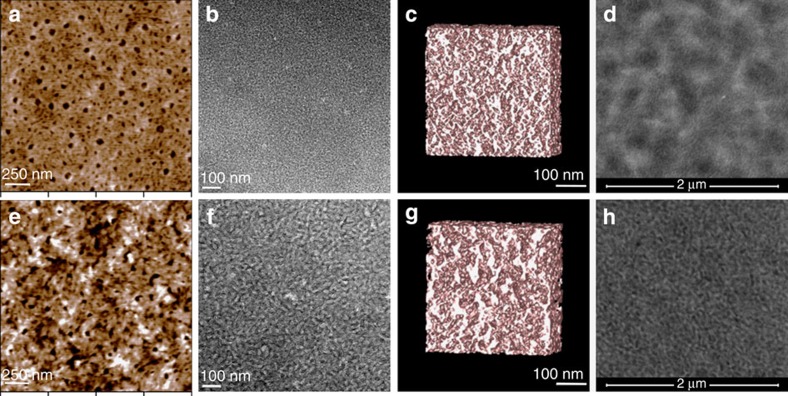
Solvent vapor annealing induced morphological changes. (**a**) AFM image shows the topography of an as-cast BTR:PC_71_BM (1:1 weight ratio) blend film. (**b**) TEM bright-field image of the as-cast film taken at a defocusing range of 3 μm. (**c**) Computer model generated from the TEM tomogram of the as-cast film. (**d**) Low-energy HAADF STEM image of the as-cast film at focus using a beam energy of 15 keV. (**e**) AFM image of the BTR:PC_71_BM blend film after THF SVA for 15 s. (**f**) TEM bright-field image of the SVA-treated film at a defocusing range of 3 μm. (**g**) Computer model of the THF SVA film. (**h**) HAADF STEM image of the blend film after SVA treatment.

To understand the effect of SVA treatment on the photovoltaic performance of BTR-based OPVs, we carried out studies on active layer morphology and the optoelectronic properties. The surface topography of the active layer was recorded by atomic force microscopy (AFM) operated in the tapping mode. Before the SVA treatment, Fig. 5a depicts a rather smooth surface, with root-mean-square roughness (*R*_rms_) of 0.61 nm. Fine crystal domains co-exist with random pinholes, which are believed to be related with the escaping of processing solvent. After a short THF SVA treatment of 15 s, the active layer exhibits a coarser surface (Fig. 5e). The *R*_rms_ value almost doubles to 1.04 nm. Transmission electron microscopy (TEM) is able to provide morphological information inside the active layer. The bright-field TEM images (Fig. 5b,f) suggest THF SVA treatment leads to larger and more well-defined domains. Because of the sharp contrast in the TEM images, we were able to obtain TEM tomograms and computer models to view the morphological change in 3D (Fig. 5c,g; Supplementary Movies). Both the TEM tomograms and their computer models show that fine-sized domains in the as-cast active layer (Supplementary Movies 1 and 2) evolve into larger domains that are inter-connected to form networks throughout the entire active layer after THF SVA for 15 s (Supplementary Movies 3 and 4). Such networks resemble ‘3D charge highways’ that are beneficial to fast charge transport. The feature size on TEM images is verified by low-energy high-angle angular dark-field scanning TEM (HAADF STEM) images (Fig. 5d,h).

The SVA treatment can be monitored by colour change of the active layer. The inset of Supplementary Fig. 13 is a digital image of the active layer before and after the THF SVA treatment. The colour of the film changed from maroon to purple upon annealing by THF vapour. Such a colour change was reflected by the change in absorption profile (Supplementary Fig. 13). There was a slight red-shift of the absorption maximum from 555 to 565 nm. Besides, the shoulder at 620 nm became more prominent, suggesting good alignment of the rod-like molecules. The absorption enhancement at 620 nm directly translated to increased photocurrent, as suggested by the EQE plot (Fig. 3c).

GIWAXS measurements were performed to understand the organization of BTR in the active layer before and after SVA. In comparison with the BTR neat film, the edge-on layered organization remains unchanged in the as-cast BTR:PC_71_BM blend film, while the π-stacking distance slightly increases to 3.80 Å and becomes randomly distributed towards the surface, as confirmed by the isotropic intensity of the corresponding peak (Supplementary Fig. 14a). The amorphous halo at *q*-range of ca. 1.25 Å^−1^ is attributed to PC_71_BM domains. SVA improves the crystallinity and surface ordering of BTR. The interlayer distance is reduced to 17.75 Å, while the π-stacking distance decreases to 3.60 Å. The random orientation of π-stacking evolves into the co-existence of both edge-on and face-on arrangements after SVA, evidenced from the π-stacking reflections at *ca*. 1.7 Å^−1^ in both *q*_xy_ and *q*_z_ directions (Supplementary Fig. 14b). Such a molecular arrangement is beneficial to 3D charge transport.

### SCLC mobilities

The hole mobility was measured using the SCLC method with a cell architecture: ITO/PEDOT:PSS/BTR:PC_71_BM/Au. Without SVA treatment, the blend film exhibited a relatively high hole mobility of 2.2 × 10^−4^ cm^2^ V^−1^ s^−1^ (Supplementary Fig. 15; Table 1). The rigid and planar backbone facilitates easy stacking and strong intermolecular interaction due to side-chain modifications. The SVA treatment substantially enhanced the mobility by one order of magnitude to 1.6 × 10^−3^ cm^2^ V^−1^ s^−1^, which is comparable to or greater than those reported for high-performing donor:acceptor blend systems^7^,^31^,^41^,^44^,^45^. The electron mobility derived from the SCLC method was also improved by one order of magnitude after SVA treatment (Supplementary Fig. 16; Table 1). Such an enhancement can be attributed to larger and more structured domains, as well as better molecular arrangement. The extremely high mobility would partially account for the high FF observed in BTR-based OPVs. However, we do not exclude other possible factors including vertical phase separation or removal of recombination centres and so on^33^.

### Solar cell stability

For practical application, solar cell-stability experiments were carried out in both air and nitrogen environments. Due to the use of active metal-like calcium for the top electrode, the unencapsulated OPVs of the thick active layer (~400 nm) degraded to almost zero efficiency within three days of storage in air (Supplementary Fig. 17; Supplementary Table 3). However, a simple encapsulation with ultraviolet (UV)-curable epoxy and thin glass slides could greatly improve the device stability in air. The OPVs degraded three times slower than that without encapsulation (Supplementary Fig. 17; Supplementary Table 4). To minimize the degradation factor due to the oxidation of electrode and further explore the stability of the active layer, one BTR-based solar cell device was stored in a glove box filled with dry nitrogen and was monitored over a time span of 30 days. The cell retained 86% of initial PCE after 7 days, and it exhibited >50% of original PCE after 30 days of storage (Supplementary Fig. 18a,b; Supplementary Table 5). Further enhancement of device stability could be achieved by improved device architecture. Average of 10 OPV cells of thick active layer of 400 nm and an additional 30-nm-thick silver top electrode/protection layer with or without encapsulation retained 92 and 86% of initial average PCE after 30 days of storage in a glove box (Supplementary Fig. 18c; Supplementary Tables 6 and 7). We believe even better stability can be obtained if the cells were properly encapsulated or inverted cell architecture was employed.

## Discussion

In summary, we present a new molecular donor, BTR, which possesses a rigid and flat backbone and a large number of flexible side chains that could work synergistically to provide excellent processability, nematic liquid crystal behaviour and optoelectronic properties. The neat BTR film exhibited hole mobilities up to 0.1 cm^2^ V^−1^ s^−1^ in OFET devices. The solution-processed single-junction BHJ solar cells based on BTR and PC_71_BM demonstrated a reproducible record efficiency of 9.3%. The blend film also supported a high FF of 77% and a high SCLC hole mobility of 1.6 × 10^−3^ cm^2^ V^−1^ s^−1^ after SVA with THF. Thick-film molecular solar cells with an active layer thickness up to 400 nm were demonstrated, showing a low thickness dependence of photovoltaic performance. Together, the results suggest BTR is an ideal candidate for printed OPVs. Moreover, enhancing the intermolecular interaction through side-chain modification is a viable way further to enhance the efficiency of molecular solar cells in excess of 10%.

## Methods

### Materials

Unless noted, all materials were reagent grade and used as received without further purification. Anhydrous solvents were prepared by drying HPLC-grade solvents using freshly activated molecular sieves.

### Synthesis of aldehyde compound 3

Synthetic route could be found in Supplementary Fig. 1. Precursor **1** and **2** were prepared using literature methods^46^,^47^. In protection of N_2_, to a dry 250-ml flask were added compound **1** (1.07 g, 1.0 mmol), compound **2** (1.05 g, 2.0 mmol), Pd_2_(dba)_3_ (46 mg, 0.04 mmol), P(*o*-tolyl)_3_ (97 mg, 0.32 mmol) and 50 ml of toluene. The reaction mixture was refluxed at 125 °C for 12 h. The reaction mixture was filtered and the crude product was purified by silica gel column chromatography (petroleum spirit 40–60 °C: dichloromethane=1:1.5, *R*_f_ 0.5) to give the product as yellow solid (830 mg, 53%). mp 146–147 °C; infrared (IR) (neat) ν 2,955, 2,923, 2,855, 1,656, 1,432, 1,225, 1,058, 820, 786 and 663 cm^−1^; ^1^H NMR (*δ*, CDCl_3_) 9.89 (s, 2 H), 7.72 (d, *J*=4.0 Hz, 2 H), 7.68 (s, 2 H), 7.23 (d, *J*=4.0 Hz, 2 H), 7.12 (s, 2 H), 7.01 (s, 2 H), 2.82 (m, 12 H), 2.65 (m, 4 H), 1.60–1.75 (m, 14 H), 1.26–1.45 (m, 52 H), 0.88 (m, 30 H); ^13^C NMR (*δ*, CDCl_3_) 182.6, 146.1, 142.6, 142.2, 141.2, 139.6, 139.0, 138.6, 137.3, 137.2, 136.9, 136.0, 135.8, 134.8, 130.3, 129.8, 129.5, 129.1, 128.3, 125.9, 123.5, 119.5, 41.7, 32.7, 32.3, 31.8, 31.6, 30.8, 30.4, 30.2, 29.8, 29.6, 29.3, 29.2, 29.1, 28.9, 28.4, 26.0, 23.1, 22.7, 22.6, 14.2 and 13.1; mass spectrometry (MS) (matrix-assisted laser desorption/ionization (MALDI)) *m*/*z* 1,630 [M].

### Synthesis of BTR molecule

To the solution of compound **3** (327 mg, 0.2 mmol) in dry chloroform (20 ml) was added rhodanine **4** (434 mg, 2.0 mmol) followed by one drop of 1,8-Diazabicyclo[5.4.0]undec-7-ene (DBU). The reaction mixture was then placed under continuous stirring at room temperature for 3 h. Reaction solvent was removed and the crude product was purified by silica gel column chromatography (petroleum spirit 40–60 °C: dichloromethane=1:1, *R*_f_ 0.8). The desired fractions were collected and the product was obtained as a purple solid (310 mg, 76%) after washing with acetone. mp 178–180 °C; IR (neat) ν 2,924, 2,855, 1,699, 1,575, 1,423, 1,327, 1,238, 1,180 and 820 cm^−1^; ^1^H NMR (*δ*, CDCl_3_) 7.83 (s, 2 H), 7.65 (s, 2 H), 7.35 (d, *J*=4.0 Hz, 2 H), 7.23 (s, 2 H), 7.20 (d, *J*=4.0 Hz, 2 H), 7.10 (s, 2 H), 7.00 (s, 2 H), 4.09 (t, *J*=7.6 Hz, 4 H), 2.81 (m, 12 H), 2.78 (m, 4 H), 1.60–1.75 (m, 18 H), 1.26–1.45 (m, 64 H), 0.88 (m, 36 H); ^13^C NMR (*δ*, CDCl_3_) 192.2, 167.5, 144.3, 141.9, 141.0, 139.5, 139.0, 138.6, 137.3, 137.2, 137.1, 135.7, 135.6, 134.8, 134.6, 130.4, 129.8, 129.7, 129.0, 128.3, 126.5, 125.0, 123.5, 120.2, 119.5, 44.9, 41.7, 32.7, 32.4, 31.8, 31.7, 31.6, 31,3, 30.8, 30.4, 30.3, 29.8, 29.7, 29.3, 29.2, 29.1, 28.9, 28.4, 27.0, 26.4, 26.0, 23.1, 22.7, 22.6, 22.5, 14.2, 13.1; MS (MALDI) *m*/*z* 2,028.7 [M]^+^. Elemental analyses calcd (%) for C114H152N2O2S14 (BTR): C, 67.41; H, 7.54; N, 1.38; O, 1.58; S, 22.10; found: C, 67.64; H, 7.65; N, 1.24; O, 1.33; S, 21.87.

### Material characterizations

IR spectra were obtained on a Perkin-Elmer Spectrum One Fourier transform infrared spectrometer and UV–visible spectra were recorded using a Cary 50 UV–visible spectrometer. Photoluminescence was measured with a Varian Cary Eclipse fluorimeter. Melting points were determined on a Büchi 510 melting point apparatus. ^1^H NMR and ^13^C NMR spectra were carried out on a 400-MHz spectrometer. All NMR data were referenced to the chloroform signal and peak multiplicity was reported as follows: s=singlet, d=doublet, t=triplet, q=quartet, p=pentet, dd=doublets of doublets, m=multiplet and br=broad). MALDI-time-of-flight MS was performed on a Bruker microflex instrument, using chloroform as solvent and dithranol as the assisted matrix. Elemental analyses were obtained commercially through Chemical & Analytical Services Pty Ltd. (Australia) an Exeter Analytical CE–440 elemental analyzer. Thermal gravimetric analysis experiments were carried out with a Mettler Toledo TGA/SDTA851e, and DSC experiments were performed on a Perkin-Elmer Sapphire DSC. CV experiments were performed at a sweep rate of 100 mV s^−1^. CVs were carried out in a three-electrode cell consisting of a glassy carbon working electrode, a platinum wire auxiliary electrode and a Ag/Ag^+^ pseudo-reference electrode. The supporting electrolyte was 0.10 M tetrabutylammonium hexafluorophosphate (Bu_4_NPF_6_) in CH_3_CN. The solutions were deoxygenated by sparging with argon prior to each scan and blanketed with argon during the scans. The glassy carbon working electrode was prepared by polishing with 5 mm alumina and washed and dried before the polymer was drop-casted on the electrode from chlorobenzene solution to form a film. Ferrocene/ferrocenium redox couple was used as the internal standard. The HOMO energy level was calculated from the onset of the oxidation potential of the polymer using the following: *E*_HOMO_=−(4.8+*E*_ox_ onset) eV.

### Crystal structure determination

Single-crystal data were collected on the MX2 beamline at The Australian Synchrotron at 100 K using a 360° rotation in steps of 1° with 1-s exposure time per step. The beamline was set to the Mo-K-α edge (17.444 keV or 0.71073 Å). Crystal data for BTR: C112 H150 N2 O2 S14, *M*=2,005.17, *T*=100.0(2) K, *λ*=0.71073 Å, Triclinic, space group *P-1*, *a=*314.257(3), *b=*20.519(4), *c=*21.795(4) Å, *α*=114.76(3)°, *β*=98.08(3)°, *γ*=02.00(3)°, *V=*5,474(2) Å^3^, *Z*=2, *D*_c_=1.217 Mg M^−3^, *μ*=0.326 mm^−1^, *F*(000)=2,152, crystal size 0.10 × 0.05 × 0.05 mm. *θ*_max_=27.47°, 88,775 reflections measured, 22,889 independent reflections (*R*_int_=0.055) the final *R*=0.11 (*I*>2*σ*(*I*), 14,445 data) and *wR*(*F*^2^)=0.3696 (all data) GOOF=1.371.

### Thin-film characterizations

To study the liquid crystal property of BTR, we used a Nikon Eclipse LV100 POM equipped with Linkam LTS350 heating and cooling stage connected to a Linkam TMS 94 temperature programmer. GIWAXS samples were prepared by spin-coating chloroform solution of BTR onto PEDOT:PSS-coated silicon substrates. The measurements were performed by means of a solid anode X-ray tube (Siemens Kristalloflex X-ray source, copper anode X-ray tube operated at 35 kV and 40 mA), Osmic confocal MaxFlux optics, X-ray beam with pinhole collimation and a MAR345 image plate detector. The beam size was 0.5 × 0.5 mm and samples were irradiated just below the critical angle for total reflection with respect to the incoming X-ray beam (~0.18°). The scattering intensity was detected on a 2D image plate (MAR345) with a pixel size of 100 μm (3,450 × 3,450 pixels). Data analysis was performed using the Datasqueeze 3.0.0 software. Atomic force microscopy images were acquired using an Asylum Research Cypher scanning probe microscope operated in tapping mode. Samples for electron microscopy were prepared by dissolving the PEDOT:PSS layer using water and transferring the floating active layer to TEM grids. TEM bright-field images were obtained by a FEI Tecnai TF30 TEM equipped a with beam-blank function. For TEM tomography, tilt series were acquired using the Xplore 3D software (FEI Company). Tomograms were recorded between −65 and +65 degrees at 2° intervals and aligned with IMOD. A 3D model rendering employed 3d mod software. Each model was generated from the aligned tomogram. The BTR donor phase was rendered in pink colour, while the PC_71_BM acceptor phase was represented by empty space in model movies. The scale bar was 100 nm in both the model movies and the tomogram movies. HAADF STEM was performed at a primary electron energy of 15 keV with a FEI Quanta 3D Microscope equipped with a HAADF STEM detector. To obtain hole and electron mobilities using SCLCs, hole-only devices (ITO/PEDOT:PSS/BTR:PC_71_BM/Au) and electron-only devices (ITO/Al/ BTR:PC_71_BM/Al) were constructed. Their dark currents were recorded by a computer-programmed Keithley 2400 source meter and then fitted by the Mott–Gurney equation. Film thickness was determined by a Veeco Dektak 150+ Surface Profiler.

### Solvent vapour annealing

SVA was conducted in a glove box filled with dried nitrogen. THF (1 ml) were injected into a 30-mm glass Petri dish. The Petri dish was closed for 1 min to let the vapour saturate the treatment chamber. Then as-cast films were attached on the backside of the Petri dish lid, which was quickly swapped with the lid covering the solvent-containing Petri dish. The film was about 1 cm above the solvent level during the SVA. After certain duration, the film was removed from the treatment chamber. The optimal duration in this study was 15 s.

### OFET fabrication and measurement

OFETs were fabricated employing two types of device configurations: bottom-gate, bottom-contact and bottom-gate, top-contact on the 300-nm-thick silicon-dioxide dielectric covering the highly doped silicon that acted as the gate electrode. The source–drain channel length (*L*) to channel width (*W*) ratio was 1:70 for bottom-contact and 1:33 for top-contact configurations. The substrates were first cleaned by sonication in acetone and isopropanol for 20 min each, then dried under nitrogen flow and dipped into a freshly prepared piranha solution (7:3 v/v H_2_SO_4_/30% H_2_O_2_) at 90 °C for 60 min. To remove residues of the piranha solution, substrates were rinsed with copious deionized water and dried under nitrogen flow. The BTR thin film was deposited via spin-coating (for top-contact OFETs) or drop-casting (bottom-contact OFETs) using 4.5 mg ml^−1^ toluene with subsequent annealing at 60 °C for 60 min to remove residual solvent. In addition, the BTR films were measured after thermal annealing at 179 and 190 °C. All the electrical measurements are performed in a glove box under nitrogen atmosphere by means of a Keithley 4200 SCS.

### Solar cell fabrication and characterization

Patterned ITO glasses were washed sequentially by detergent, deionized water, acetone and 2-propanol in an ultrasonication bath and UV/ozone-treated. PEDOT:PSS (Clevios P VP AI 4083) was spin-coated at 6.000 r.p.m. and then baked at 140 °C for 10 min in air. The substrates were transferred to a glove box filled with dried nitrogen, where a chloroform solution of the donor–acceptor blend was spin coated at various spin rates on top of the substrate. The best PCE was achieved by spin coating 20 mg BTR and 20 mg PC_71_BM in 1 ml of chloroform at 1,000 r.p.m. The resulting film thickness was around 250 nm. Thicker or thinner films were produced by varying the solution concentration and spin rate from 1.6 to 2.0% and from 600 to 2,000 r.p.m., respectively. To obtain high photovoltaic performance, the films were solvent vapour annealed by THF. Then they were transferred to a thermal evaporator where 40 nm Ca and 100 nm aluminium were deposited through a shadow mask (active area was 0.11 cm^2^) at a base pressure of 4 × 10^−7^ mbar. The solar cells were encapsulated under nitrogen by UV-curable epoxy (Epotek OG112-6 by Epoxy Technology Inc.) and cover glass. They were tested in air with a computer-programmed Keithley 2400 source meter under a Newport Oriel class A solar similar, which simulated the AM1.5 solar irradiance with energy density of 98 mW cm^−2^. For accurate measurement and to avoid oxidation during testing, a batch of optimized solar cells were brought to a third-party research institute, Solar Energy Research Institute of Singapore, where the cells were tested under nitrogen and 1 SUN (100 mW cm^−2^) condition provided by a 1 kW solar simulator (Sun 2000 Solar Simulator by ABET Technologies) that was calibrated by a silicon reference cell (Fraunhofer ISE) and a Schott visible-colour glass-filtered (KG5 colour filtered) Si diode (Hamamatsu S1133). OPVs made in Australia followed the same fabrication procedures, and were tested at Clayton Laboratories of Commonwealth Scientific and Industrial Research Organisation, where a 1-kW Oriel solar simulator with an AM 1.5G filter was used as the light source.

## Author contributions

K.S. fabricated the solar cell device and characterized the film and device properties using POM, XRD, AFM, TEM, HAADF STEM and SCLC. Z.X. designed and performed materials synthesis and characterization including NMR, MS, CV and UV–vis absorption spectra for solution and thin film. S.L. scaled up the synthesis, grew single crystals and measured DSC, TGA, PL and solubility. W.Z. and W.P. fabricated OFET, measured and analysed OFET, 2D-XRD and GIWAXS data. E.H. constructed TEM tomograms and computer models. J.M.W. solved the crystal structure using XRD acquired by R.M.W. at Australian Synchrotron. J.S. reproduced the OPV results. K.S., Z.X. and S.L. prepared the manuscript. All authors discussed the results and commented on the manuscript. J.O., W.W.H.W., A.B.H. and D.J.J. supervised the project and revised the manuscript.

## Additional information

**Accession codes**: The crystallographic information file for BTR has been deposited with Cambridge Crystallographic Data Centre, and signed to CCDC code 1029304.

**How to cite this article**: Sun, K. *et al.* A molecular nematic liquid crystalline material for high-performance organic photovoltaics. *Nat. Commun.* 6:6013 doi: 10.1038/ncomms7013 (2015).

## Supplementary Material

Supplementary Figures and Supplementary Tables Supplementary Figures 1-20, Supplementary Tables 1-8

Supplementary Movie 1 Tilt series of as-cast BTR:PC_71_BM blend film with thickness of 160 nm. Scale bar 100 nm.

Supplementary Movie 2 Computer model of as-cast BTR:PC_71_BM blend film with thickness of 160 nm. Scale bar 100 nm.

Supplementary Movie 3 Tilt series of SVA-treated BTR:PC_71_BM blend film with thickness of 100 nm. Scale bar 100 nm.

Supplementary Movie 4 Computer model of SVA-treated BTR:PC_71_BM blend film with thickness of 100 nm. Scale bar 100 nm.

## Figures and Tables

**Table 1:** Photovoltaic parameters of BTR:PC_71_BM BHJ solar cells fabricated and tested under different conditions

***J***_**sc**_ **(mA cm**^−**2**^**)**	***V***_**oc**_ **(V)**	**FF (%)**	**PCE (%)**	***R***_s_* **(Ω** **cm**^**2**^**)**	***R***_**sh**_† **(MΩ cm**^**2**^**)**	***μ***_**h**_‡ **(cm**^**2**^ **V**^−**1**^ **s**^−**1**^**)**	***μ***_**e**_‡ **(cm**^**2**^ **V**^−**1**^ **s**^−**1**^**)**
*As-cast*§							
11.64	0.96	47	5.2	14.0	5.5	2.2 × 10^−4^	3.5 × 10^−4^
(11.20±0.51)	(0.96±0.01)	(42±3)	(4.5±0.4)	—	—		
							
*THF SVA*§							
13.52	0.89	73	8.7	2.4	42		
[12.16]	[0.90]	[76]	[8.3]	2.2	6.4		
(13.17±0.43)	(0.89±0.01)	(71±2)	(8.3±0.2)	—	—	1.6 × 10^−3^	9.6 × 10^−3^
							
*THF SVA*||							
13.90	0.90	74	9.3	2.7	15		
[13.40]	[0.90]	[77]	[9.3]	1.9	20		
(13.61±0.16)	(0.89±0.01)	(74±1)	(8.9±0.2)	—	—		

FF, fill factor; PCE, power conversion efficiency; SCLC, space-charge-limited current; SVA, solvent vapour annealing; THF, tetrahydrofuran.

Data in brackets show the cell with the highest FF. Data in parentheses are average results out of 40+ encapsulated devices tested in air or 28 non-encapsulated devices tested in nitrogen.

^*^Series resistance of the device.

^†^Shunt resistance of the device.

^‡^Charge mobility values obtained from SCLC experiments.

^§^Cells were tested in air with encapsulation, illumination intensity was 98 mW cm^−2^.

^||^Cells without encapsulation were tested in a glove box filled with dry nitrogen; illumination intensity was 100 mW cm^−2^.
