# Supplementary material for: A molecular nematic liquid crystalline material for high-performance organic photovoltaics
Source: Nat Commun. 2015 Jan 14;6:6013. doi: 10.1038/ncomms7013 (PMC4309440; doi:10.1038/ncomms7013)
Supplement: Supplementary Figures and Supplementary Tables — Supplementary Figures 1-20, Supplementary Tables 1-8 [file ncomms7013-s1.pdf]

Supplementary Information

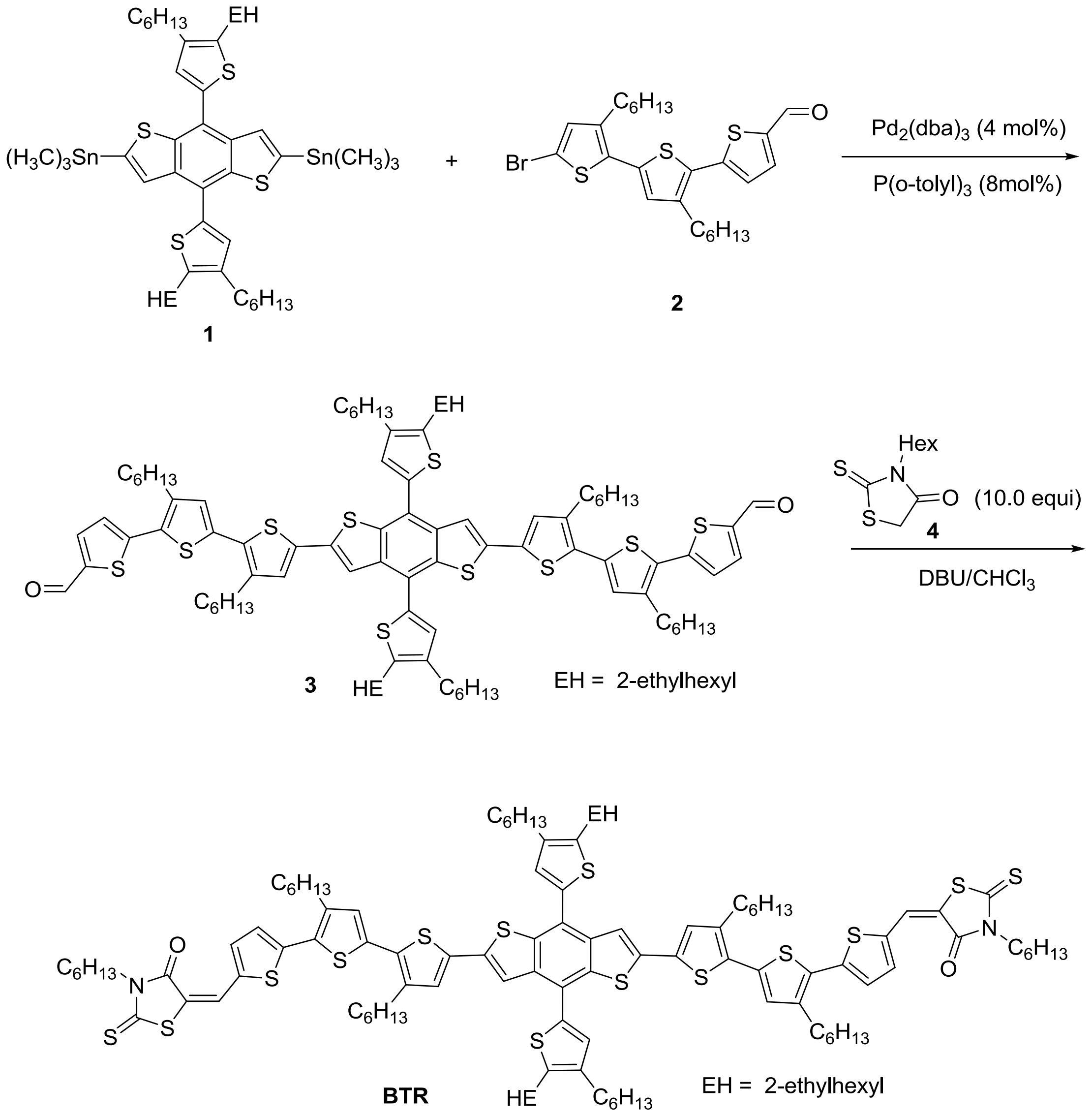

Supplementary Figure 1 | Synthetic route of BTR.

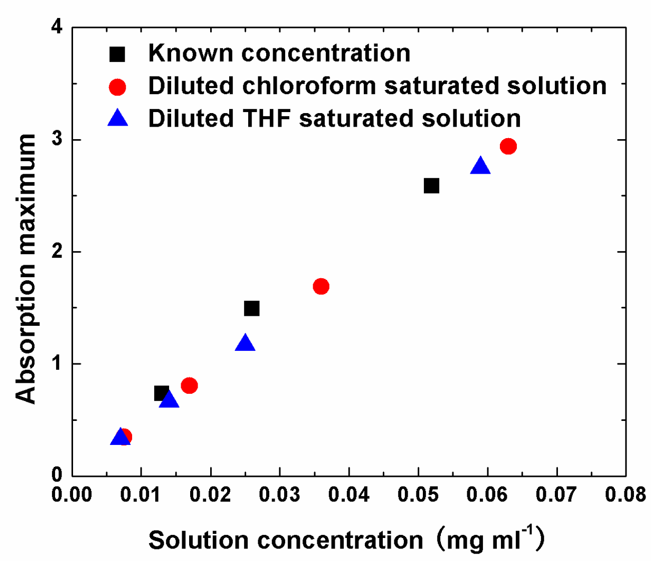

Supplementary Figure 2 | Derivation of solubility of BTR in chloroform or THF. Plot of absorption maximum vs. solution concentration for BTR molecules in chloroform of known concentration and diluted solutions of BTR/chloroform or BTR/THF saturated solutions.

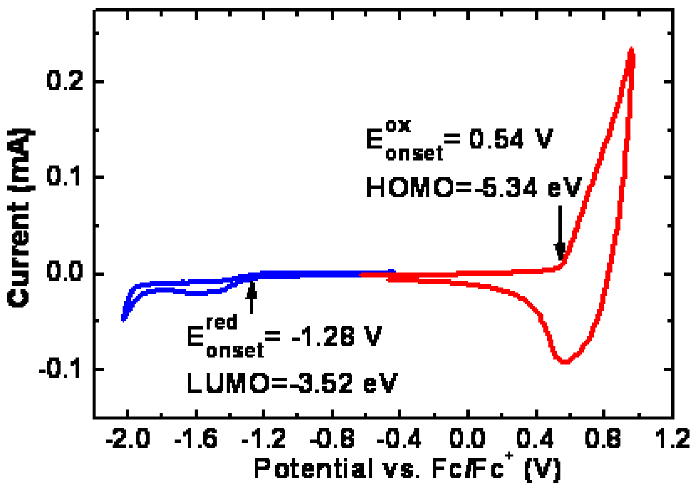

Supplementary Figure 3 | Cyclic voltammogram of BTR thin film. Reference to Fc/Fc^+ redox potential at a sweep speed of 100 mV s^{-1}.

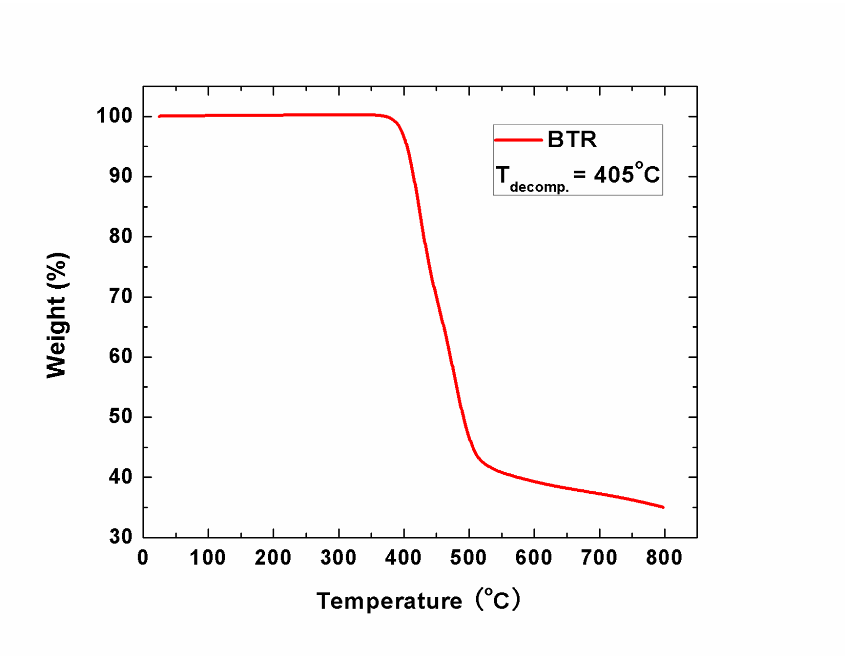

Supplementary Figure 4 | TGA plot for BTR molecules. Sample was heated in nitrogen at a rate of 10 $^{o}C\ s^{-1}$.

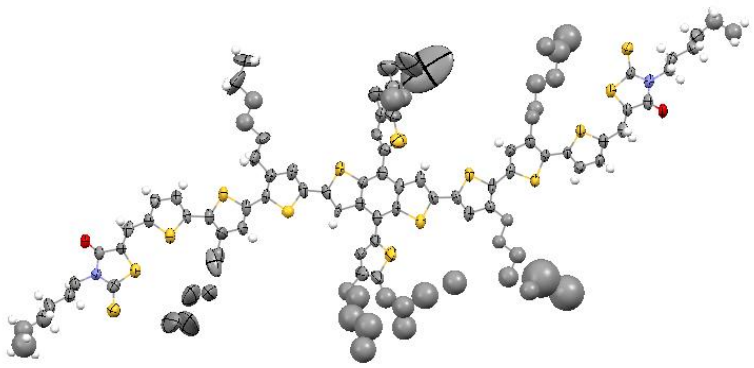

Supplementary Figure 5 | Ortep diagram of BTR molecule in a single crystal structure as determined by X-ray crystallography. X-ray quality single crystals were obtained by slow diffusion of 2-propanol into saturated BTR solution in dichloromethane at room temperature. The extended backbone is well resolved, however, many of the side chain carbon atoms are extensively disordered, particularly in the regions of the 2-ethylhexyl substituents, which are disordered due to the combination of high thermal motion and the co-crystallization of all possible diastereomeric forms of the 2-ethylhexyl groups in the crystal.

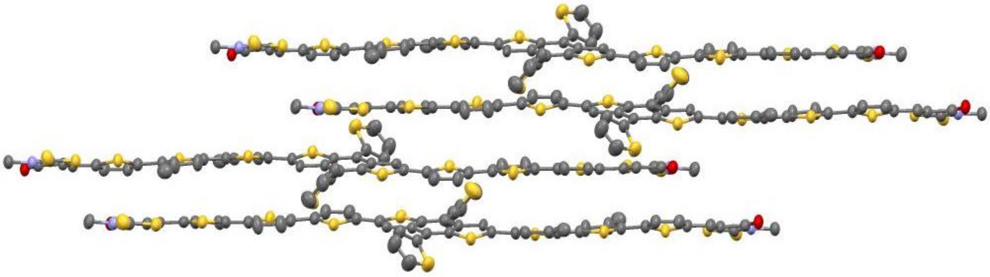

Supplementary Figure 6 | Side view of BTR aggregation. BTR molecules aggregate into π-stacked dimers by further π-stacking, with an average interplaner separation of 3.62Å.

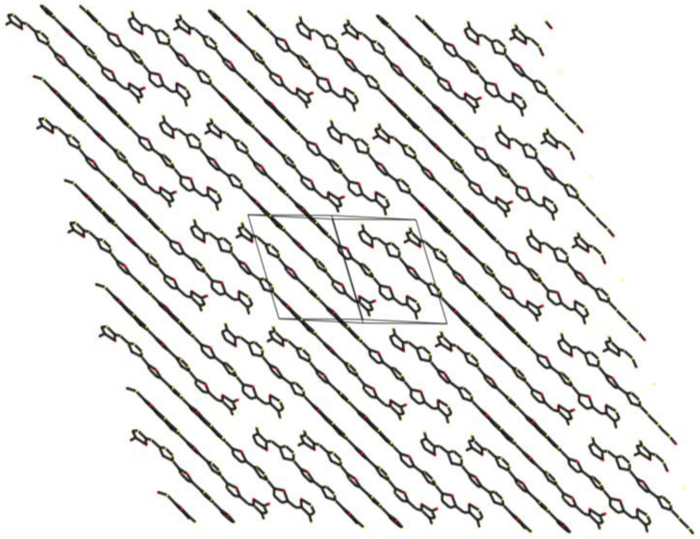

Supplementary Figure 7 | Crystal packing of BTR backbones in the (011) plane. The π-stacking extends in a brick-wall type pattern within the crystallographic (011) plane.

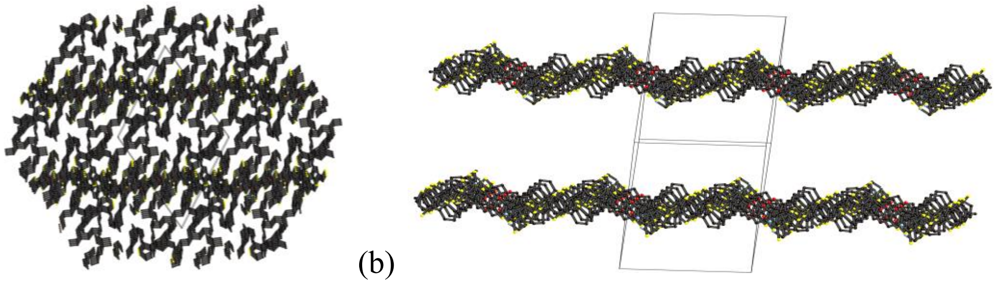

(a) (b)

Supplementary Figure 8 | Layers of the π-stacked BTR molecules. (a) with and (b) without showing the alkyl side chains. The parallel planes of the π-stacked BTR molecules are held together by weak van der Waals interactions between the interpenetrating alkyl side chains and are arranged down the zy direction

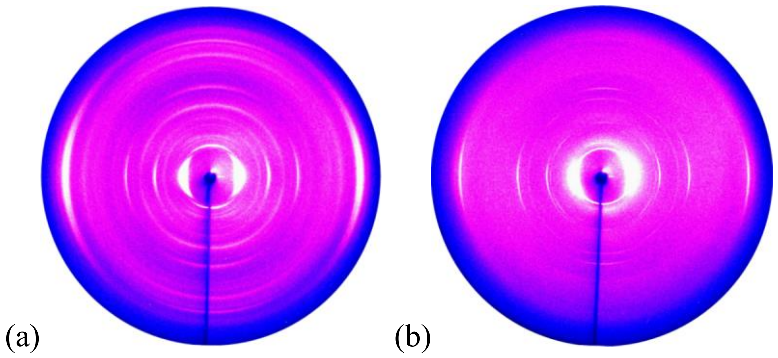

Supplementary Figure 9 | 2D-WAXS of BTR filaments. Measurements were done at (a) 30 °C and (b) 179 °C.

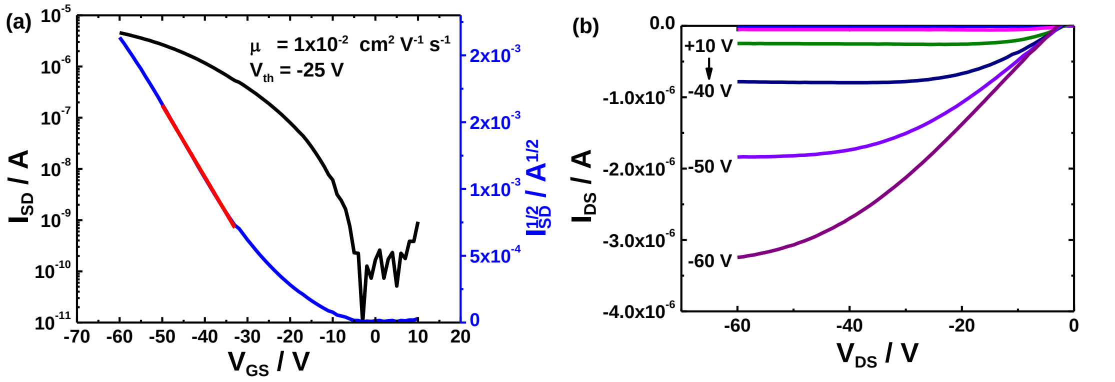

Supplementary Figure 10 | Mobility measurement using spin-cast OFET devices. (a) Transfer characteristics in semi-log plot and in square-root plot as a function of gate voltage, and (b) output characteristics of a *p*-channel OFET device containing BTR thin film deposited by spin coating BTR/toluene solution on silicon substrate treated by piranha solution with top contact bottom gate configuration of electrodes after thermal annealing at 179 °C.

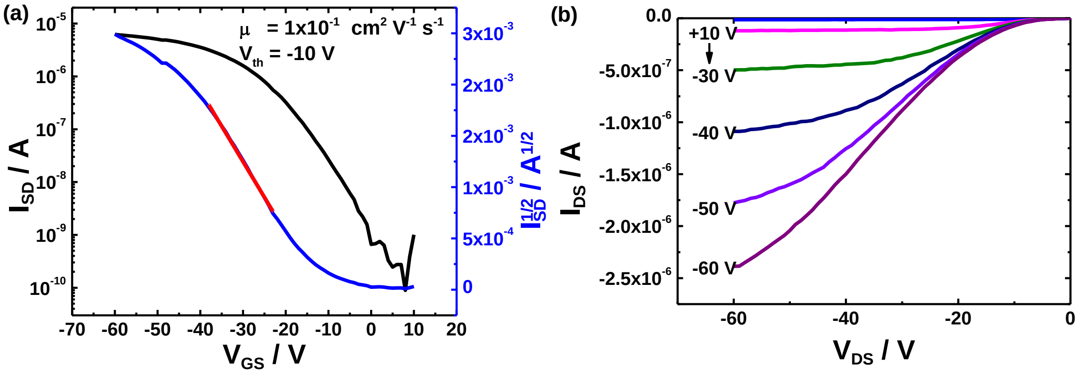

Supplementary Figure 11 | Mobility measurement using drop-cast OFET devices. (a) Transfer characteristics in semi-log plot and in square-root plot as a function of gate voltage, and (b) output characteristics of a *p*-channel OFET device containing BTR thin film deposited by drop casting BTR/toluene solution on silicon substrate treated by piranha solution with bottom contact bottom gate configuration of electrodes after thermal annealing at 190 ^{o}C.

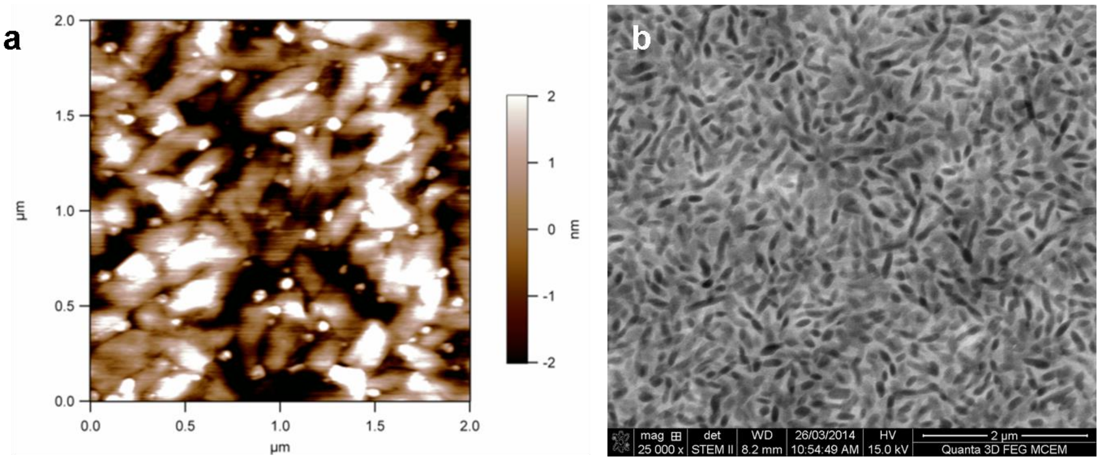

Supplementary Figure 12 | Thermal annealing effect on BTR:PC_{71}BM blend film. a, AFM image shows the topography of the BTR:PC_{71}BM blend film after thermal annealing. The root-mean-square roughness is 1.69 nm for the 2×2 μm image. **b,** HAADF STEM image taken at 15 keV beam energy of the BTR:PC_{71}BM blend film after thermal annealing. The scale bar is 2 μm. Thermal annealing for the active layer was carried out in nitrogen at 110 ^{o}C for 1 min. The AFM and HAADF STEM images revealed over-growth of the donor and acceptor phases.

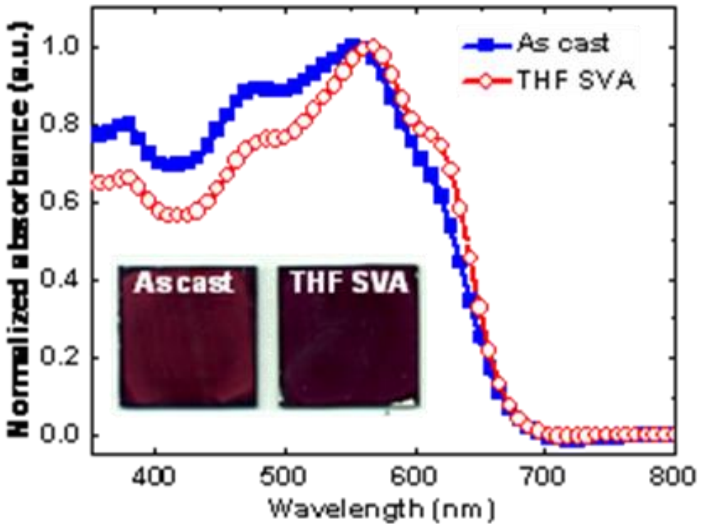

Supplementary Figure 13 | Optical property change due to SVA. Normalized UV-vis spectra of the blend films before and after THF SVA treatment for 15 s. Inset: A digital picture showing the color change of the blend film due to SVA treatment.

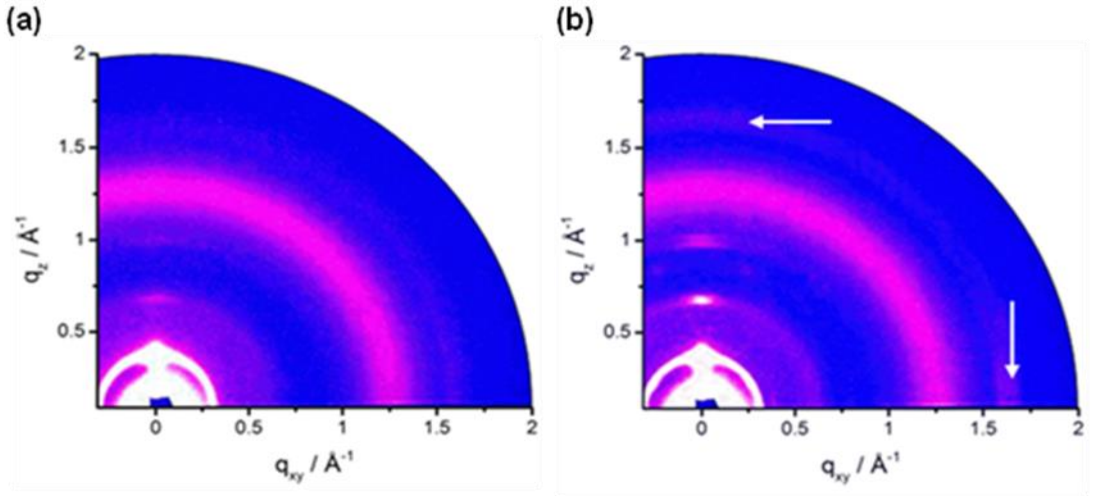

Supplementary Figure 14 | Structual change due to SVA effect. GIWAXS of (a) as-cast BTR:$PC_{71}BM$ film and (b) after THF solvent vapor annealing (π-stacking reflection of BTR is indicated by arrows).

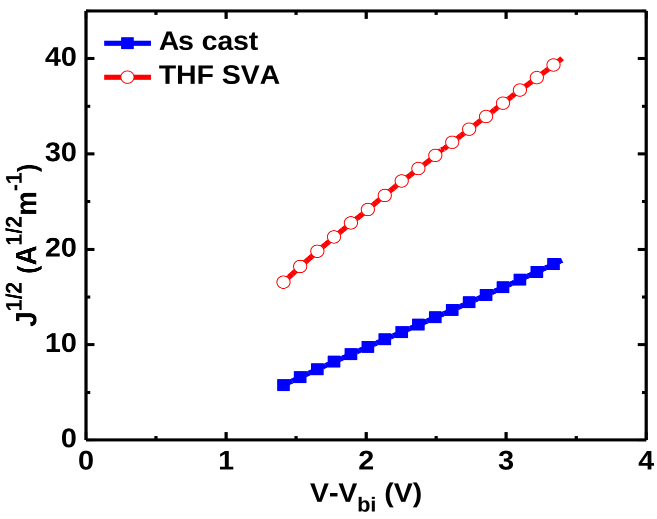

Supplementary Figure 15 | Hole mobility derived from SCLC method. Plots of $J^{0.5}$ vs. effective bias of the dark curves of hole-only devices, ITO/PEDOT:PSS/BTR:$PC_{71}BM$/Au. The active layer has a thickness of 290-320 nm.

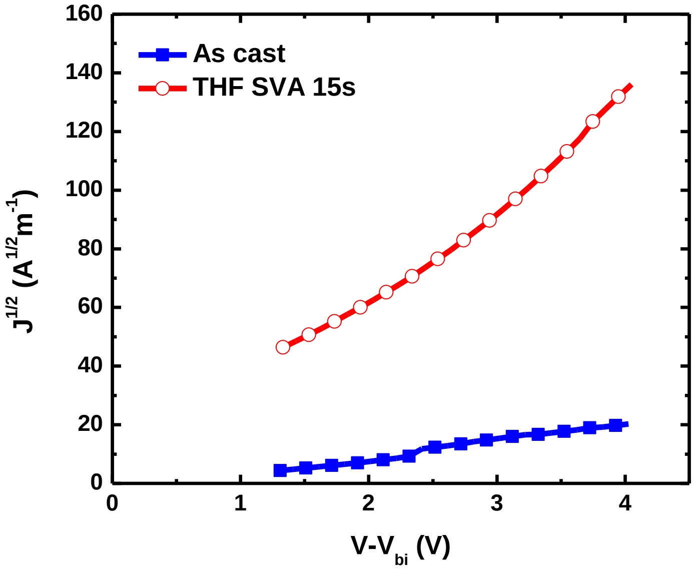

Supplementary Figure 16 | Electron mobility change by SVA. Plots of $J^{0.5}$ vs. effective bias of the dark curves of electron-only devices, ITO/Al/BTR:$PC_{71}BM$/Al. The active layer has a thickness of 290-310 nm.

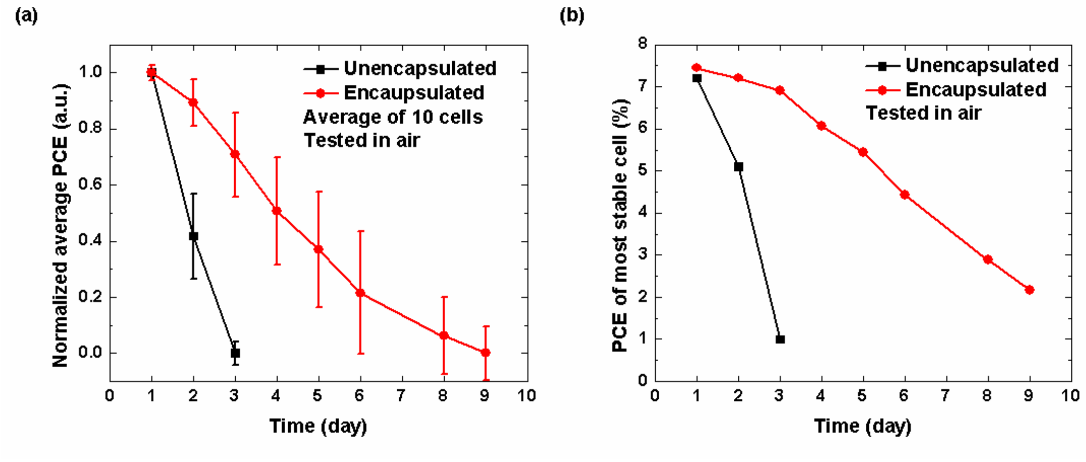

Supplementary Figure 17 | Stability of OPVs stored in air. Stability plots of BTR-based molecular solar cells without and with encapsulation tested in air for (a) average of 10 cells and (b) the most stable cell.

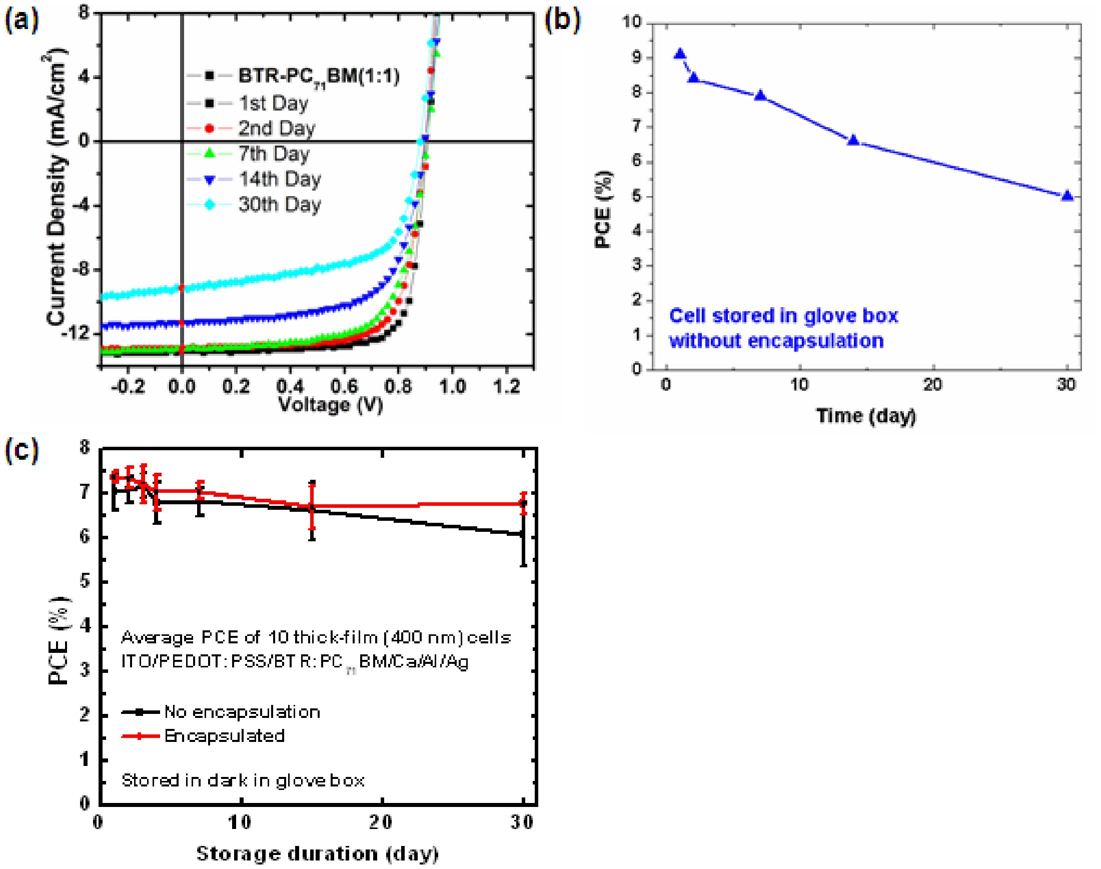

Supplementary Figure 18 | Stability of BTR-based OPV cells stored in glove box. (a) *J-V* plot and stability plot of (b) an optimized unencapsulated BTR:$PC_{71}BM$ OPV cell and (c) average PCE of 10 cells of thick active layer (thickness ~ 400 nm) and an extra 30 nm-thick Ag protection layer, with or without encapsulation, stored and tested in nitrogen environment over a duration of 30 days.

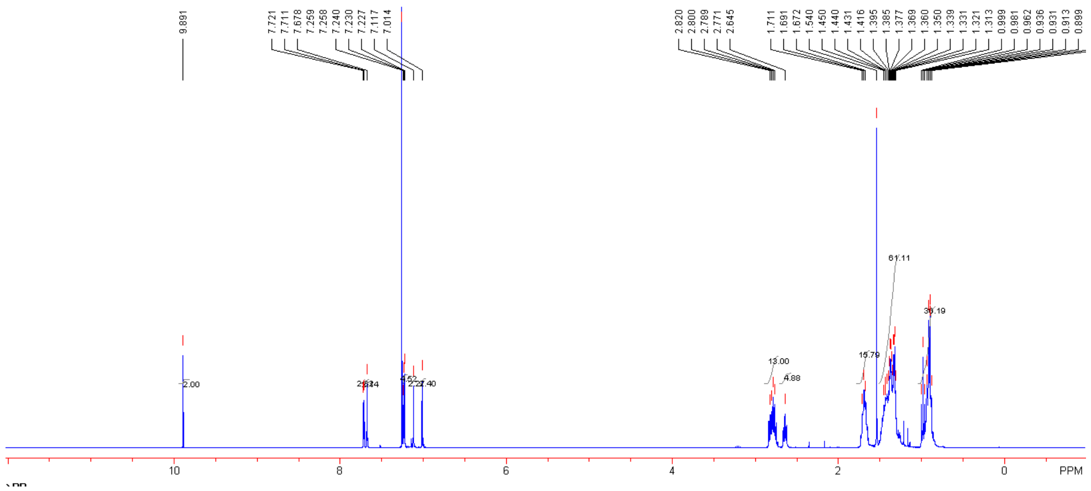

^{1}H NMR spectrum of compound **3 (**$CDCl_3$, 400 MHz, 25°C).

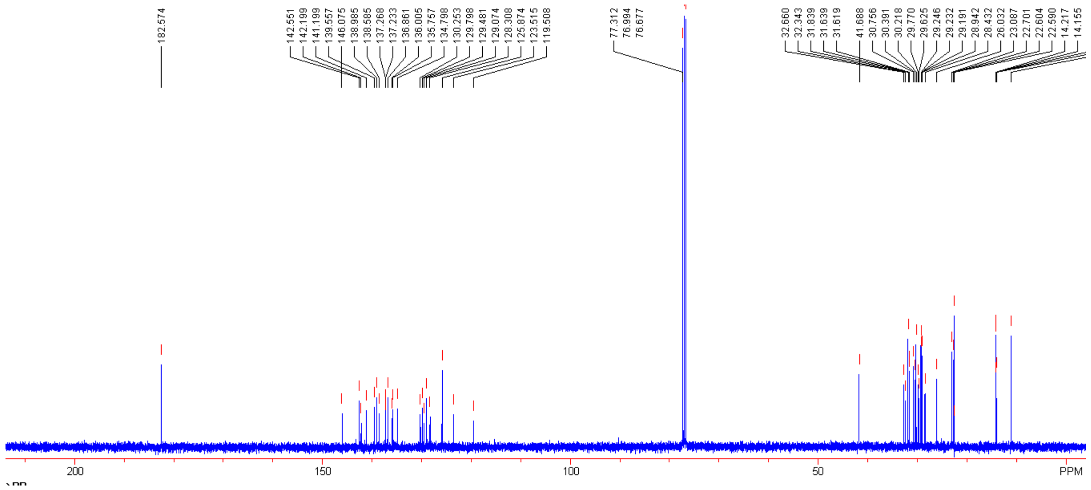

^{13}C NMR spectrum of compound **3** ($CDCl_3$, 100 MHz, 25°C).

Supplementary Figure 19 | NMR spectra of compound 3.

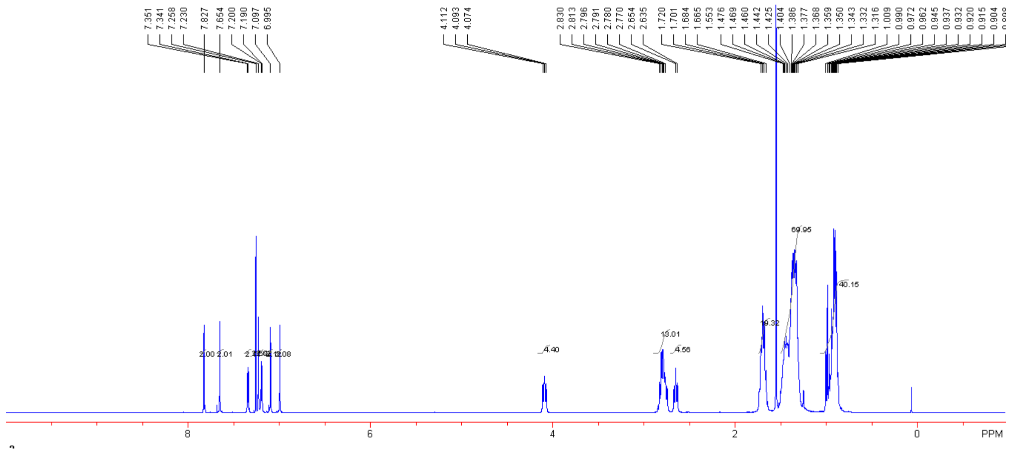

^{1}H NMR spectrum of compound **BTR** ($CDCl_3$, 400 MHz, 25°C).

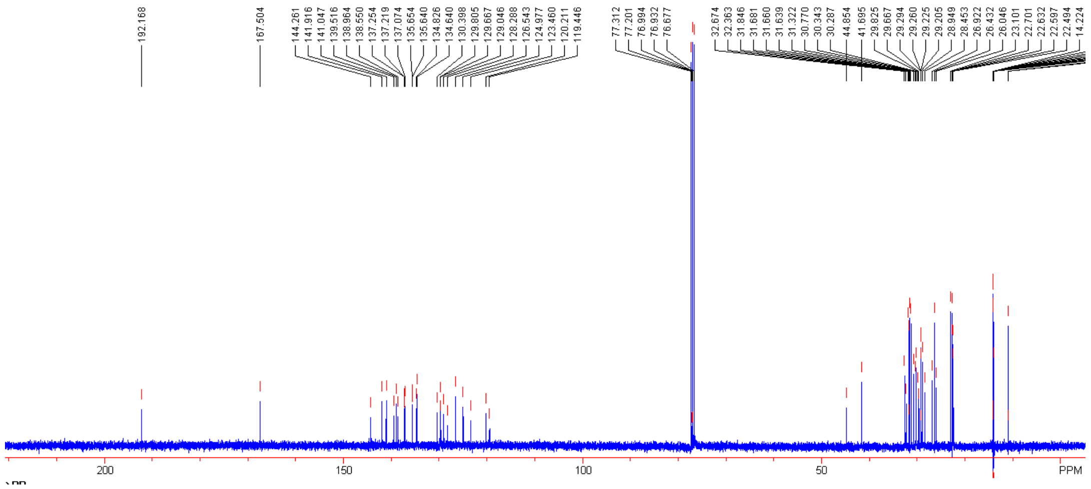

^{13}C NMR spectrum of compound **BTR** ($CDCl_3$, 100 MHz, 25°C).

Supplementary Figure 20 | NMR spectra of BTR.

Supplementary Table 1 | Summary of optical and electrochemical properties of BTR molecule.

Solution λ_{max} (nm)	Film λ_{max} (nm)	Optical frontier orbital energy gap (eV)	HOMO (eV)	LUMO (eV)	Electrochemical HOMO-LUMO gap (eV)
523	572, 620	1.82	-5.34	-3.52	1.82

Supplementary Table 2 | Average photovoltaic parameters of at least 8 optimized BTR:$PC_{71}BM$ BHJ solar cells with different active layer thicknesses. The cells were encapsulated and tested in air.

Thickness (nm)	J_{sc} (mA cm^{-2})	V_{oc} (V)	FF	PCE (%)
**80**	10.26±0.71	0.89±0.01	0.74±0.01	6.8±0.5
**110**	10.47±0.80	0.89±0.01	0.75±0.01	6.9±0.5
**170**	11.48±0.25	0.89±0.01	0.74±0.01	7.6±0.1
**250**	13.17±0.43	0.89±0.01	0.71±0.02	8.3±0.2
**330**	12.71±0.21	0.89±0.01	0.69±0.01	7.9±0.2
**350**	13.04±0.40	0.88±0.01	0.69±0.01	8.0±0.2
**400**	12.71±0.21	0.88±0.01	0.68±0.01	7.6±0.2

Supplementary Table 3 | Average photovoltaic parameters of 10 BTR:$PC_{71}BM$ BHJ solar cells of thick active film (400 nm). The cells were stored in air without encapsulation and tested daily.

No. of days	J_{sc} (mA cm^{-2})	V_{oc} (V)	FF	PCE (%)
**1**	12.02±0.44	0.86±0.00	0.70±0.01	7.3±0.2
**2**	5.11±1.92	0.86±0.01	0.70±0.01	3.1±1.1
**3**	0.16±0.50	0.69±0.11	0.27±0.16	0.1±0.3

Supplementary Table 4 | Average photovoltaic parameters of 10 BTR:$PC_{71}BM$ BHJ solar cells of thick active film (400 nm). The cells were encapsulated and tested daily in air.

No. of days	J_{sc} (mA cm^{-2})	V_{oc} (V)	FF	PCE (%)
**1**	12.11±0.17	0.86±0.00	0.70±0.01	7.3±0.2
**2**	11.15±0.91	0.86±0.00	0.69±0.01	6.6±0.6
**3**	9.30±1.85	0.86±0.01	0.68±0.01	5.4±1.1
**4**	7.02±2.26	0.86±0.01	0.68±0.01	4.1±1.4
**5**	5.52±2.56	0.86±0.00	0.67±0.02	3.2±1.5
**6**	3.77±2.59	0.84±0.07	0.60±0.15	2.2±1.6
**8**	2.18±1.72	0.76±0.17	0.53±0.20	1.2±1.0
**9**	1.43±1.22	0.74±0.19	0.52±0.18	0.8±0.7

Supplementary Table 5 | Photovoltaic parameters of an optimized BTR:$PC_{71}BM$ BHJ solar cells during storage in a glove box. The cells were not encapsulated and tested in nitrogen.

No. of days	J_{sc} (mA cm^{-2})	V_{oc} (V)	FF	PCE (%)
**1**	13.05	0.90	0.77	9.1
**2**	12.90	0.90	0.72	8.4
**7**	12.90	0.90	0.68	7.9
**14**	11.30	0.88	0.66	6.6
**30**	9.10	0.88	0.62	5.0

Supplementary Table 6 | Average photovoltaic parameters of 10 OPV cells with cell architecture ITO/PEDOT:PSS/BTR:$PC_{71}BM$/Ca/Al/Ag during storage in a glove box. The cells were not encapsulated and tested in nitrogen.

No. of days	J_{sc} (mA cm^{-2})	V_{oc} (V)	FF	PCE (%)
**1**	12.43±0.27	0.85±0.03	0.66±0.02	7.04±0.40
**2**	12.24±0.56	0.86±0.00	0.67±0.02	7.10±0.29
**3**	12.36±0.40	0.86±0.00	0.68±0.02	7.18±0.28
**4**	11.96±0.45	0.84±0.07	0.68±0.04	6.80±0.46
**7**	11.79±0.64	0.87±0.00	0.67±0.01	6.82±0.32
**15**	11.25±0.99	0.87±0.00	0.68±0.05	6.60±0.65
**30**	10.87±1.32	0.86±0.01	0.65±0.03	6.09±0.71

Supplementary Table 7 | Average photovoltaic parameters of 10 OPV cells with cell architecture ITO/PEDOT:PSS/BTR:$PC_{71}BM$/Ca/Al/Ag during storage in a glove box. The cells were encapsulated and tested in nitrogen.

No. of days	J_{sc} (mA cm^{-2})	V_{oc} (V)	FF	PCE (%)
**1**	12.73±0.27	0.86±0.00	0.67±0.01	7.36±0.13
**2**	12.73±0.36	0.86±0.00	0.67±0.01	7.33±0.23
**3**	12.62±0.80	0.86±0.00	0.67±0.02	7.20±0.42
**4**	12.35±0.66	0.86±0.00	0.66±0.01	7.03±0.40
**7**	12.71±0.29	0.87±0.00	0.64±0.01	7.05±0.18
**15**	12.03±0.74	0.87±0.00	0.64±0.02	6.70±0.47
**30**	12.49±0.21	0.86±0.01	0.63±0.01	6.77±0.24

Supplementary Table 8 | Crystal data and structure refinement for BTR.

Identification code	shelx	
Empirical formula	C112 H150 N2 O2 S14	
Formula weight	2005.17	
Temperature	100.0(2) K	
Wavelength	0.71073 Å	
Crystal system	Triclinic	
Space group	P -1	
Unit cell dimensions	a = 14.257(3) Å	α= 114.76(3)°.
	b = 20.519(4) Å	β= 98.08(3)°.
	c = 21.795(4) Å	γ = 102.00(3)°.
Volume	5474(2) Å^3	
Z	2	
Density (calculated)	1.217 Mg/m^3	
Absorption coefficient	0.326 mm^{-1}	
F(000)	2152	
Crystal size	0.10 x 0.05 x 0.05 mm^3	
Theta range for data collection	1.064 to 27.470°.	
Index ranges	-18<=h<=18, -26<=k<=26, -28<=l<=28	
Reflections collected	88775	
Independent reflections	22889 [R(int) = 0.0547]	
Completeness to theta = 25.242°	92.6 %	
Absorption correction	None	

Refinement method	Full-matrix least-squares on F^2
Data / restraints / parameters	22889 / 92 / 961
Goodness-of-fit on F^2	1.371
Final R indices [I>2sigma(I)]	R1 = 0.1101, wR2 = 0.3356
R indices (all data)	R1 = 0.1474, wR2 = 0.3696
Extinction coefficient	n/a
Largest diff. peak and hole	1.276 and -1.161 e.Å^{-3}
